# Regulatory Effects of “Straw-Nitrogen Fertilizer” on Maize Yield Enhancement

**DOI:** 10.3390/plants15060962

**Published:** 2026-03-20

**Authors:** Yuchen Zhang, Mingxue Ye, Jinman Mei, Qiulai Song, Xiaochen Lyu, Chunmei Ma

**Affiliations:** 1 College of Agriculture, Northeast Agricultural University, Harbin 150030, China; zyc18646306513@163.com (Y.Z.); yemx0121@163.com (M.Y.); 15376385800@163.com (J.M.); 2 Institute of Crop Cultivation and Tillage, Heilongjiang Academy of Agricultural Sciences, Harbin 150086, China; sql142913@163.com

**Keywords:** straw return, nitrogen fertilization, continuous maize cropping, nutrient uptake and utilization, soil nitrogen cycling

## Abstract

To elucidate the regulatory mechanisms underlying the interaction between straw return and nitrogen (N) fertilization on yield formation, nutrient uptake, and soil N cycling in a continuous maize cropping system, a two-year positioning experiment was conducted. The study established two straw treatments (S0: 0 g/box; S1: 84 g/box) combined with three N levels (N0: 0 g/box; N1: 1.24 g/box; N2: 2.47 g/box). (The box refers to the cylinder used for planting maize.) The responses of maize yield, plant nutrient accumulation and partitioning, fertilizer-derived N ratio, nitrogen fertilizer use efficiency (NUE), and soil microenvironment were analyzed. Results indicated that under N1 conditions, straw return had a negligible effect on crop growth and yield formation. Conversely, under N2 conditions, straw return significantly enhanced maize yield and promoted the accumulation of N, phosphorus (P), and potassium (K) in plant tissues. ^15^N isotope tracing revealed a novel mechanism: rather than significantly altering direct fertilizer nitrogen use efficiency, straw return improved crop yield primarily by elevating indigenous soil N content and boosting the activities of N-transforming enzymes, thereby beneficially altering the ultimate environmental fate of the fertilizer N. Furthermore, straw return significantly boosted the activities of enzymes involved in N transformation and optimized the soil microenvironment. Collectively, straw return coupled with increased N application (specifically the S1N2 treatment) significantly maximizes maize yield, providing a theoretical basis for rational straw utilization and N management.

## 1. Introduction

Maize (*Zea mays* L.) ranks as a globally pivotal cereal crop, second only to wheat and rice. Extensively cultivated for food, feed, and industrial applications [1], it plays a critical role in agricultural production and global food security. Nitrogen (N) fertilization is a primary determinant of maize yield. As a crucial driver of global agricultural productivity [2], N input directly governs maize growth and yield formation. However, less than half of the applied N is typically taken up by the crop; the remainder either accumulates in the soil or is lost to the environment. Such N losses from agricultural systems exacerbate environmental pollution [3].

Although crop residues cause greenhouse gas (GHG) emissions in the short term [4], they serve as a valuable food source through direct or indirect rotation practices [5]. Specifically, straw incorporation directly inputs substantial nutrients into the soil while indirectly stimulating microbial activity. This suggests that beyond direct nutrient provision, straw return enhances plant nutrient uptake in continuous cropping systems by ameliorating the soil environment [6,7]. Furthermore, straw represents a unique slow-release nutrient management strategy distinct from conventional slow-release fertilizers, where nutrient release kinetics are governed by its intrinsic and surface structural properties [8]. Concurrently, the organic compounds in straw can elevate soil nutrient levels and pH buffering capacity, thereby enhancing nutrient retention and sustaining long-term soil fertility [9,10].

Straw return not only enhances soil nitrogen (N) supply capacity but also promotes plant N uptake and utilization to a certain extent. The increase in soil N content under straw return is closely associated with soil enzyme activities [11], while changes in the soil microenvironment influence inorganic N storage and availability [12]. Compared to chemical fertilization alone, this approach is more conducive to the sustainable development of agroecosystems.

Rational N fertilization under straw return conditions plays a critical role in improving NUE, ensuring yield stability, and protecting the environment. Straw is rich in essential macro- and micronutrients, which are vital for enhancing soil fertility and maintaining ecosystem functions [13]. Straw incorporation, accompanied by the action of soil N-transforming enzymes, increases soil N stocks and accelerates N immobilization [13], subsequently releasing N required for crop growth. However, maize produces a higher biomass yield than other crops, and the return of straw with a C:N ratio exceeding 25:1 creates a soil environment rich in carbon but deficient in nitrogen for microorganisms [14,15]. The competition for N between straw decomposition and crop uptake often leads to a transient decline in soil N during the initial stage of decomposition. Although soil N levels gradually recover as degradation proceeds, this temporary N immobilization can disrupt soil N supply and crop N acquisition [16]. Applying N fertilizer can mitigate the increase in the soil C:N ratio caused by straw return, alleviate soil N limitation, and relieve the competition for N between straw decomposition and crop uptake.

Therefore, this study used a ^15^N isotope tracing technique to monitor labeled N fertilizer under controlled N application rates and straw return regimes. The experiment analyzed the interaction effects of straw return and N fertilization on maize N, phosphorus (P), and potassium (K) accumulation, NUE, soil total nitrogen, and soil N-transforming enzyme activities of soil nitrate reductase (S-NR), soil nitrite reductase (S-NiR), soil urease (S-UE), as well as their ultimate impact on maize yield. The objective was to elucidate the regulatory mechanisms of the straw-N interaction on plant nutrient status, fertilizer N fate, soil N dynamics, and enzymatic activity. Although the transient decline in soil N during initial straw decomposition is well-documented, the precise mechanisms underlying the fate of fertilizer nitrogen and the responses of specific soil nitrogen-transforming enzymes in continuous high-yield maize systems remain largely unresolved. A thorough understanding of how supplemental nitrogen alters the ultimate partitioning of fertilizer nitrogen to bridge this gap is still lacking. These findings provide a theoretical basis for precise straw management and optimized N fertilization strategies in high-yield and high-efficiency maize cultivation.

## 2. Results

### 2.1. Nutrient Assimilation by Maize Plants

**Figure 1:**
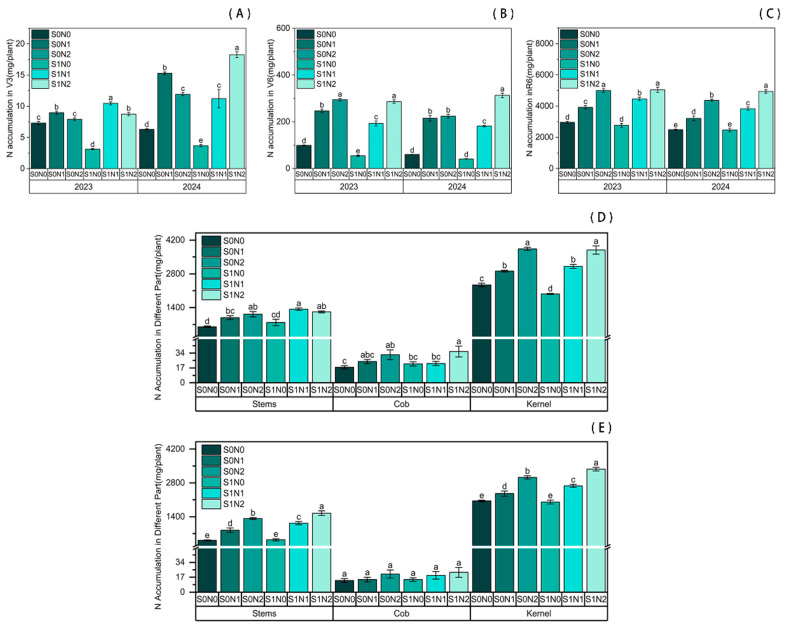
Nitrogen accumulation in maize plants under different treatments. (**A**) V3 stage; (**B**) V6 stage; (**C**) R6 stage. (**D**,**E**) Nitrogen accumulation in different organs at the R6 stage in 2023 (**D**) and 2024 (**E**). Different lowercase letters indicate significant differences between treatments at *p* < 0.05.

Our study demonstrated that straw return coupled with nitrogen fertilization significantly enhanced N accumulation in maize as Figure 1. Under identical straw return conditions, N application promoted N accumulation at the V3, V6, and R6 stages. Both N1 and N2 treatments exhibited significantly higher N accumulation compared to the non-N control (N0). Notably, at the early growth stage (V3), N accumulation was higher in the N1 treatment than in N2. However, this trend reversed at the V6 stage and across different organs at the R6 stage, where the N2 treatment consistently outperformed N1.

Under equivalent N fertilization levels, straw return significantly enhanced N uptake. At the V3 stage, N accumulation in the S1N2 treatment increased by 8.75% and 55.56% across the two years compared to S0N2. At the jointing stage (V6) in 2024, S1N2 showed a 39.70% increase relative to S0N2. By maturity (R6), N accumulation in the S1N2 treatment was significantly higher (by 12.89%) than in S0N2 in 2024. Specifically, N accumulation in grains, stems/leaves, and cobs under S1N2 increased significantly by 11.24%, 16.70%, and 9.13%, respectively, compared to S0N2.

**Figure 2:**
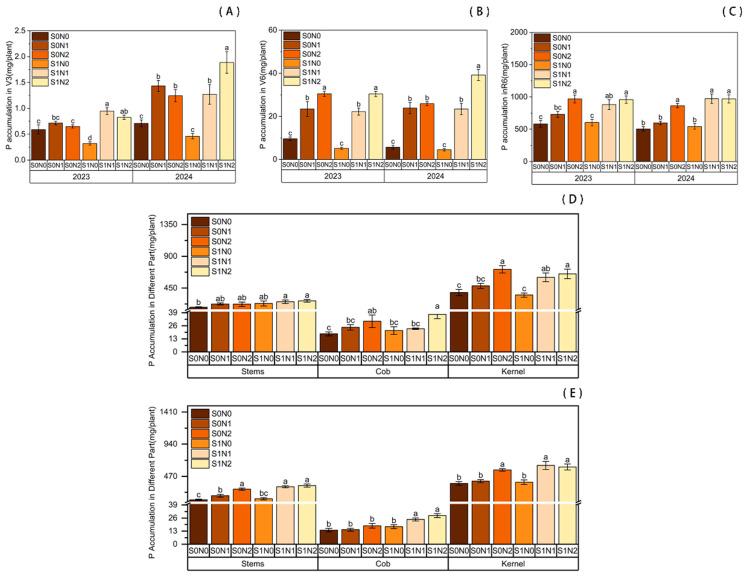
Phosphorus accumulation in maize plants under different treatments. (**A**) V3 stage; (**B**) V6 stage; (**C**) R6 stage. (**D**,**E**) Phosphorus accumulation in different organs at the R6 stage in 2023 (**D**) and 2024 (**E**). Different lowercase letters indicate significant differences between treatments at *p* < 0.05.

Furthermore, straw return combined with nitrogen fertilization significantly enhanced phosphorus accumulation in maize as shown in Figure 2. Under identical straw return conditions, N application promoted P accumulation at the V3, V6, and R6 stages. Both N1 and N2 treatments exhibited significantly higher P accumulation compared to the N0 control. Notably, during the early growth stages (V3 and V6), P accumulation was higher in the N1 treatment than in N2. However, at the R6 stage, P accumulation in different organs was significantly higher in the N2 treatment than in N1.

Under equivalent N levels, straw return significantly facilitated P uptake. At the V3 stage, P accumulation in the S1N2 treatment increased by 27.13% and 51.85% over the two years, respectively, compared to S0N2. At the jointing stage (V6) in 2024, S1N2 showed a 51.30% increase relative to S0N2. By maturity (R6), P accumulation in the S1N2 treatment was significantly higher (by 12.06%) than in S0N2 in 2024. Specifically, P accumulation in grains and cobs under S1N2 increased significantly by 7.37% and 54.95%, respectively, compared to S0N2.

**Figure 3:**
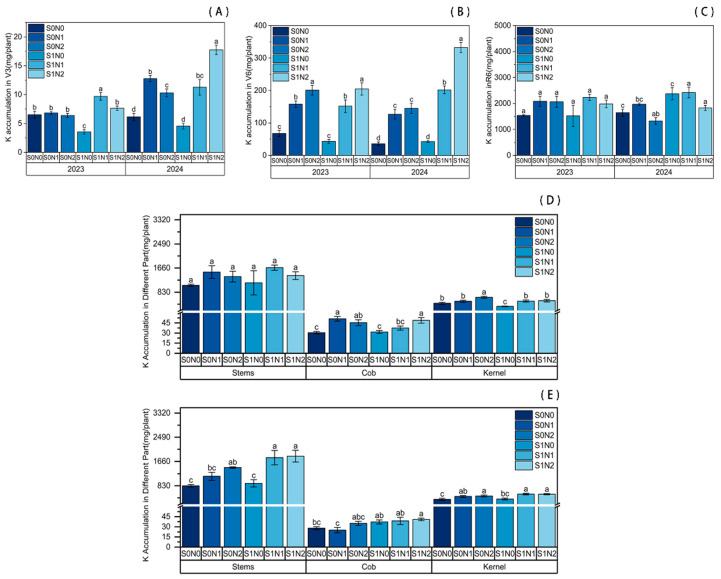
Potassium accumulation in maize plants under different treatments. (**A**) V3 stage; (**B**) V6 stage; (**C**) R6 stage. (**D**,**E**) Phosphorus accumulation in different organs at the R6 stage in 2023 (**D**) and 2024 (**E**). Different lowercase letters indicate significant differences between treatments at *p* < 0.05.

Results indicated that straw return combined with nitrogen fertilization significantly enhanced potassium accumulation in maize as shown in Figure 3. Under identical straw return conditions, N application promoted K accumulation at the V3, V6, and R6 stages. Both N1 and N2 treatments yielded significantly higher K accumulation compared to the N0 control. Specifically, in 2024, the S0N2 treatment increased K accumulation by 67.37% at the V3 stage and by 305.06% at the jointing stage compared to S0N0. Furthermore, at the V6 stage, K accumulation was significantly higher in the N2 treatment than in N1.

Under equivalent N conditions, straw return significantly facilitated K uptake. At the V3 stage, K accumulation in the S1N2 treatment increased by 19.76% and 72.30% over the two years, respectively, compared to S0N2. At the jointing stage in 2024, S1N2 exhibited a 129.06% increase relative to S0N2. By maturity, total K accumulation in the S1N2 treatment was significantly higher (by 37.70%) than in S0N2 in 2024. Specifically, K accumulation in grains, stems and leaves, and cobs under S1N2 significantly increased by 13.24%, 26.40%, and 17.01%, respectively, compared to S0N2.

### 2.2. Nitrogen Derived from Fertilizer and Nitrogen Use Efficiency

**Figure 4:**
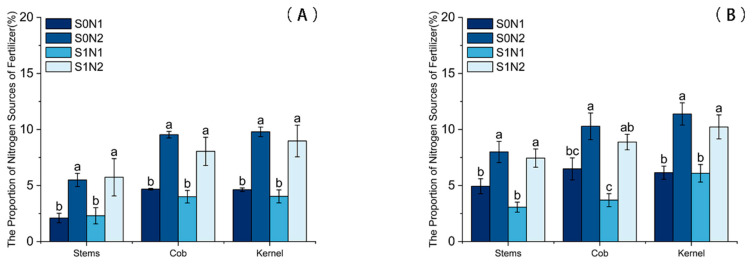
Percentage of nitrogen derived from fertilizer (Ndff) in maize plants under different treatments. (**A**) 2023; (**B**) 2024. Different lowercase letters indicate significant differences between treatments at *p* < 0.05.

Nitrogen fertilization significantly increased the percentage of nitrogen derived from fertilizer (Ndff) in maize as Figure 4. Under identical straw return conditions, increasing the N application rate significantly elevated Ndff, with both N1 and N2 treatments showing significantly higher values than the N0 control. Specifically, in 2024 under straw return conditions, the Ndff values in grains and cobs for the S1N2 treatment were 67.84% and 140.0% higher, respectively, than those for S1N1.

Conversely, under equivalent N fertilization rates, straw return resulted in a decrease in Ndff. In 2023, grain Ndff in the S1N2 treatment decreased by 8.32% compared to the non-straw control (S0N2). This trend persisted in 2024, where straw return (S1N2) significantly reduced the Ndff in grains, stems and leaves, and cobs by 10.18%, 6.88%, and 13.67%, respectively, relative to the S0N2 treatment.

**Figure 5:**
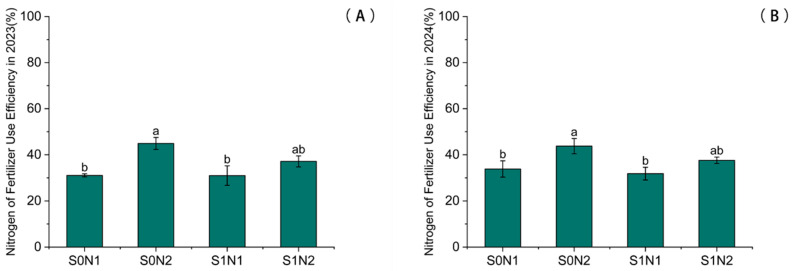
Impact of different treatments on nitrogen use efficiency (NUE) in maize. (**A**) 2023; (**B**) 2024. Different lowercase letters indicate significant differences between treatments at *p* < 0.05.

Under identical straw return conditions, increasing nitrogen (N) application significantly elevated the NUE. In 2023, as shown in Figure 5A, the NUE under the N2 treatment increased significantly by 44.47% and 19.86% compared to the N1 treatment under S0 and S1 conditions, respectively. Similarly, in 2024, as shown in Figure 5B, the N2 treatment showed increases of 29.21% and 18.16% relative to the N1 treatment. Conversely, under equivalent N application rates, straw return tended to diminish the NUE. In 2023, the NUE in the S1N2 treatment was significantly lower (by 17.29%) than that in the S0N2 treatment. This inhibitory effect persisted into 2024, with the S1N2 treatment exhibiting a 14.04% reduction in NUE compared to the S0N2 treatment.

### 2.3. Soil Microenvironment and Nutrients

Our results indicated that straw return combined with nitrogen fertilization significantly enhanced the soil total nitrogen (TN) content as Table 1. N application primarily elevated TN levels in the surface soil layer. In 2023, within the 0–15 cm soil layer, the S0N2 and S1N2 treatments increased TN by 9.91% and 8.03% compared to their respective controls (S0N0 and S1N0). Under equivalent N fertilization rates, straw return significantly further improved soil TN content, with the S1N2 treatment showing a more pronounced effect than S0N2. Specifically, in the 0–15 cm layer, the S1N2 treatment exhibited significant increases of 21.31% and 33.61% in 2023 and 2024, respectively, relative to S0N2. In the deeper soil layer (15–30 cm), the S1N2 treatment also showed consistent improvements, with TN levels increasing by 10.08% and 15.45% over the two years compared to S0N2.

Nitrogen fertilization exerted a substantial stimulatory effect on soil enzyme activities. In the 0–15 cm soil layer during 2023 as shown in Table 2, soil nitrate reductase (S-NR), nitrite reductase (S-NiR), and urease (S-UE) activities in the S0N2 treatment were 113.51%, 42.01%, and 53.41% higher, respectively, than those in the S0N0 control. Similarly, S-UE activity in the S1N2 treatment significantly increased by 43.62% compared to S1N0. In 2024 as shown in Table 3, the S0N2 treatment consistently exhibited significantly higher activities across all three enzymes in the 0–15 cm layer relative to the S0N0 control.

Regarding straw management, the S1N2 treatment showed a more pronounced enhancement in enzyme activities compared to S0N2 in 2023. Specifically, in the 0–15 cm layer, S-NR, S-NiR, and S-UE activities in S1N2 significantly increased by 32.91%, 13.46%, and 34.64%, respectively, relative to S0N2. In the deeper soil layer (15–30 cm), straw return (S1N2) notably elevated S-NR and S-UE activities by 59.68% and 24.63%, respectively, compared to S0N2. This stimulatory effect persisted in 2024, where the S1N2 treatment significantly outperformed S0N2. In the surface layer (0–15 cm), S-UE activity in S1N2 increased by 17.91% relative to S0N2, while significant enhancements in both S-NR and S-UE activities were observed in the 15–30 cm soil layer under straw return.

### 2.4. Maize Yield and Correlation Analysis

Nitrogen application markedly improved ear characteristics. In 2023 as shown in Table 4, the S0N2 treatment exhibited substantial enhancements in kernels per ear and ear length, which increased by 38.36% and 18.04%, respectively, compared to the S0N0 control.

Regarding straw management, in 2024, the S1N2 treatment recorded a 0.67% higher 100-kernel weight relative to S0N2, while representing a significant elevation of 29.23% compared to the S1N0 treatment. Furthermore, the number of kernels per ear under S1N2 was significantly higher (by 9.24%) than that under the S0N2 treatment.

**Figure 6:**
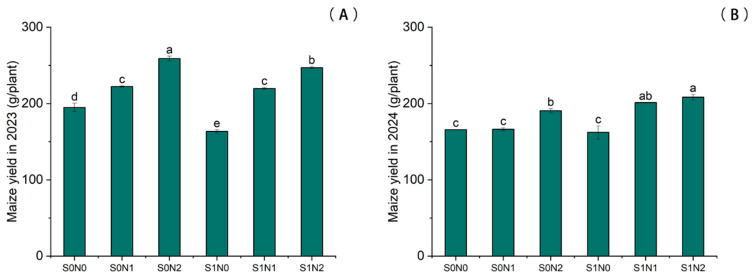
Maize grain yield during the 2023–2024 growing seasons. (**A**) 2023; (**B**) 2024. Different lowercase letters indicate significant differences between treatments at *p* < 0.05.

The integration of straw return and nitrogen (N) fertilization significantly enhanced maize grain yield as shown in Figure 6. Under identical straw management, N application significantly boosted yield, with treatments following the order of N2 > N1 > N0. For instance, in the S0 treatments of 2023, S0N2 exhibited a 32.93% increase in yield compared to the S0N0 control. Similarly, in 2024, the S0N2 treatment showed a 14.90% increase relative to S0N0.

Under equivalent N fertilization rates, straw return significantly further improved yield performance. In 2024, the yield per plant under the S1N2 treatment was significantly higher (by 9.38%) than that under S0N2. Furthermore, the grain yield of the S1N1 treatment increased significantly by 21.05% compared to the S0N1 treatment.

**Figure 7:**
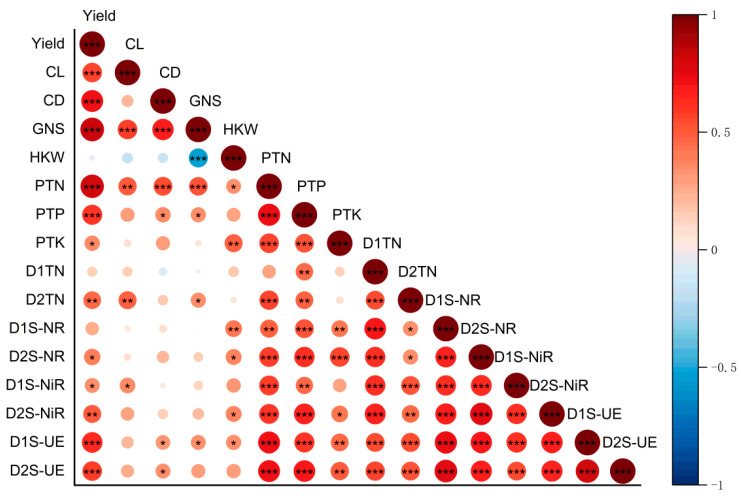
Correlation analysis between maize yield, yield components, and soil microenvironmental factors. Yield, maize grain yield; CL, cob length; CD, cob diameter; GNS, grain number per cob; HKW, hundred kernel weight; PTN, plant total nitrogen at the maturity stage; PTP, plant total phosphorus at the maturity stage; PTK, plant total potassium at the maturity stage; TN, soil total nitrogen; S-NiR, soil nitrite reductase; S-UE, soil urease; S-NR, soil nitrate reductase. D1 and D2 represent the 0–15 cm and 15–30 cm soil layers, respectively. * *p* ≤ 0.05, ** *p* ≤ 0.01, *** *p* ≤ 0.001. Circles of different sizes represent the size of the data.

As shown in Figure 7, maize yield (Yield) was highly significantly and positively correlated with plant total nitrogen (PTN) and plant total phosphorus (PTP). Among these, the correlation with PTN was the strongest, indicating that nitrogen uptake is the core determinant of yield. Simultaneously, yield was positively correlated with plant total potassium (PTK). Furthermore, yield exhibited significant positive correlations with soil urease activity (D1S-UE and D2S-UE) in both the 0–15 cm and 15–30 cm soil layers, demonstrating that urease, as a key enzyme in nitrogen mineralization, directly regulates yield formation. Additionally, yield was positively correlated with total nitrogen (D2TN) and nitrite reductase activity (D2S-NiR) in the subsoil (15–30 cm). Regarding yield components, grain number per spike (GNS) was significantly and positively correlated with PTN, while 100-kernel weight (HKW) was positively correlated with soil nitrate reductase (D1S-NR and D2S-NR) and PTK.

## 3. Discussion

### 3.1. Plant Nutrient Accumulation and Grain Yield

The accumulation of nitrogen (N), phosphorus (P), and potassium (K) in plants serves as the material foundation for crop yield, reflecting the regulatory efficacy of straw return and N fertilization on continuous maize production. Current findings indicate that the influence of straw return on nutrient uptake follows a “suppression–promotion” pattern—specifically, early-stage inhibition followed by late-stage facilitation—which was most pronounced under the S1N2 treatment. This initial inhibition is primarily attributable to the soil carbon-to-nitrogen (C/N) ratio imbalance during straw decomposition. As highlighted by Hua et al. [16] and Zhao et al. [17], the high C/N ratio of maize straw disrupts the inherent soil C/N equilibrium, triggering a “competition for nitrogen” between microbial decomposition and crop uptake. Consequently, straw return significantly reduces shoot biomass and N accumulation during the early and mid-growth stages [17,18]. In this study, however, the S1N2 treatment effectively mitigated this negative effect. This improvement stems from the fact that supplemental N fertilization optimizes the soil C/N ratio, satisfying the N demand for straw decomposition and alleviating microbial N immobilization, thereby accelerating the transition of nutrient cycling from soil sequestration to crop assimilation [19].

As the growth stages progressed, the “late-stage promotion” effect of straw return became evident. Yang et al. [20] posited that although straw induces N immobilization initially, these sequestered nutrients are re-released during the later growth stages, particularly the grain-filling period. This process compensates for the limitations of chemical fertilizers, which offer high immediacy but short duration, ensuring sustained nutrient accumulation throughout the entire life cycle [20]. In this context, straw acts as a slow-release nutrient reservoir, whose mineralization peak precisely coincides with the peak nutrient demand during maize grain filling [21]. Furthermore, the S1N2 treatment exhibited a significant surge in K accumulation. This can be explained by the release characteristics of straw as a “biological K pool,” which markedly elevates soil K levels and facilitates its uptake by the plant [22].

Grain yield is a critical metric for evaluating the synergy between straw return and rational N fertilization. Based on the yield data from 2023 and 2024, the S1N2 treatment achieved the highest output. Lv [23] noted that straw return alone (S1N0) often leads to N immobilization during the seedling stage due to C/N imbalance, which subsequently hinders spike differentiation. Similarly, Zhang et al. [24] found that maize yield depends on the synergistic enhancement of grain number per ear and 1000-kernel weight. Under S1N0 conditions, the early competition for N often results in insufficient supply during the critical spike differentiation period, leading to floret abortion and reduced grain numbers [24]. Conversely, the S1N2 treatment in this study regulated the soil C/N balance through sufficient N input, thereby reducing early-stage stress and ensuring nutrient availability during key growth stages. This is consistent with findings by Gao and the long-term experiments by Zhang [25,26], which suggest that straw return transitions from a “nutrient competitor” to a “nutrient contributor” only when N application reaches an adequate threshold, significantly increasing grain number and grain-filling density. Collectively, the combined application of organic and inorganic fertilizers maintains a continuous nutrient supply and avoids the diminishing marginal returns often associated with exclusive chemical fertilization, thereby ensuring stable and high yields in continuous cropping systems [26].

### 3.2. Fate of Fertilizer Nitrogen and Nitrogen Use Efficiency

The proportion of Ndff and NUE exhibited a significant upward trend with increasing N application levels. Interestingly, straw return did not exert a significant impact on either Ndff or NUE. However, given the significant yield enhancement observed under the S1N2 treatment, these results suggest that straw return improves productivity without substantially altering the crop’s direct uptake of fertilizer-derived nitrogen. This indicates that straw return avoids inducing nitrogen loss while simultaneously boosting yield by altering the fate of fertilizer nitrogen. According to Li et al. [27], the carbon sources introduced by straw facilitate the microbial immobilization of fertilizer nitrogen, effectively channeling it into soil organic nitrogen pools, such as microbial biomass nitrogen (MBN).

Using ^15^N labeling, Ding et al. [28] confirmed that rational N fertilization significantly accelerates the mineralization of nitrogen within the straw and the activation of indigenous soil nitrogen. Consequently, yield increases are more heavily reliant on the assimilation of these “non-fertilizer nitrogen sources” rather than a simple increase in direct fertilizer nitrogen uptake. Although this transformation may not elevate immediate fertilizer nitrogen recovery efficiency in the short term, it effectively mitigates nitrogen losses [29], serving as a long-term reservoir for late-season crop growth.

The yield improvement is primarily driven by the synchronized release of nitrogen from both the soil pool and the decomposing straw. Zhang et al. [30] utilized ^15^N tracing to demonstrate that straw return facilitates the partitioning of fertilizer nitrogen into MBN and particulate organic nitrogen (PON); while this biological immobilization limits immediate fertilizer nitrogen uptake, it successfully curtails nitrogen losses and enhances soil nitrogen retention [30]. Wang et al. [21] further noted that straw return significantly expands the storage of mineral-associated organic nitrogen. Under high-yield conditions, crops tend to assimilate more “soil-derived nitrogen” provided by mineralization and decomposition rather than relying exclusively on chemical fertilizers. This influx of exogenous nitrogen creates a dilution effect on the proportion of fertilizer nitrogen within the plant, leading to an increase in total nitrogen accumulation while nitrogen use efficiency remains stable [31]. Furthermore, Li et al. [32] confirmed that straw return improves water use efficiency by optimizing soil porosity and water-holding capacity. Superior hydraulic conditions enable crops to produce more biomass at equivalent nitrogen uptake levels, achieving a synergistic promotion of yield through optimized water and fertilizer coupling [33].

### 3.3. Soil Nutrients and the Microenvironment

Current results demonstrate that straw return and nitrogen (N) application levels significantly influence soil nutrient content and N-transforming enzyme activities, thereby altering the soil microenvironment with distinct vertical differentiation across soil layers. The elevation in soil total nitrogen (TN) is primarily driven by the C/N interaction mechanism between straw and N fertilizer. As noted by Li [27], sole straw return (S1N0) often triggers microbial N scavenging from the soil due to the carbon-rich but nitrogen-poor nature of the residue. Conversely, the addition of exogenous N in the S1N2 treatment rebalances the soil C/N ratio, accelerating straw decomposition and humification. This process facilitates the transformation of both organic N from straw and inorganic N from fertilizer into a stable soil TN pool. This “fertilizer-driven carbon sequestration and carbon-mediated N retention” cycle significantly enhances the soil TN storage capacity compared to other treatments, providing a sustainable nutrient buffer reservoir for maize growth [27,29]. Returning straw to the field combined with increased nitrogen fertilizer application prevents the early seedling inhibition commonly caused by nitrogen immobilization under straw incorporation conditions. By integrating organic residues with inorganic fertilizers, this approach reduces environmental nitrogen loss and avoids the diminishing returns associated with sole chemical fertilizer application. It ensures the nutrient supply for critical grains, thereby providing a highly applicable strategy for maintaining high and stable yields in global continuous maize cropping systems.

Soil enzyme activities serve as sentinel indicators of the soil microenvironment and nutrient transformation capacity. In this study, the S1N2 treatment significantly boosted the activities of key enzymes, including S-UE and S-NR. Yang et al. [33] pointed out that the enhancement of S-UE activity stems from a dual stimulatory effect: straw provides abundant carbon sources as energy substrates for enzymatic reactions, while N fertilizer acts as an inductive substrate. Highly active urease accelerates the hydrolysis of N fertilizer, ensuring a rapid supply of ammonium N (NH_4_^+^-N) in the soil [33]. Simultaneously, research by Tang [34] suggests that appropriate N fertilization activates S-NR, promoting the assimilation and utilization of soil nitrate N (NO_3_^−^-N), thereby mitigating the risk of leaching associated with excessive N accumulation [33].

The optimization of the soil microenvironment plays a pivotal role in modulating these enzymatic activities. Li [32] confirmed that straw return fosters a coordinated hydrothermal microenvironment by improving soil porosity and water retention, which provides an ideal medium for the stability of enzyme proteins and substrate diffusion [31]. Furthermore, the S1N2 treatment helps maintain a balanced activity of S-NiR. Jiang et al. [35] found that an optimal “straw-nitrogen” ratio maintains an appropriate redox potential, preventing excessive denitrification enzyme activity (such as S-NiR) and subsequent N losses caused by localized anaerobic conditions [35]. Rather than merely increasing nutrient quantity through fertilization, the S1N2 treatment drives the transformation of N species by stimulating key enzyme activities and expanding the TN pool. This mechanism explains the fundamental reason for the simultaneous improvement in maize yield and nutrient use efficiency under this treatment [36]. As maize is a globally pivotal crop, returning straw to the field combined with increased nitrogen fertilizer is highly conducive to the sustainable development of agroecosystems worldwide by protecting the environment while ensuring yield stability.

## 4. Materials and Methods

### 4.1. Experimental Materials

This study was conducted during the 2023–2024 growing seasons, based on a long-term micro-plot experiment established in 2016. The micro-plots were constructed using bottomless rigid plastic cylinders (0.30 m diameter, 0.33 m height). Each cylinder was filled with approximately 26 kg of soil to a depth of 0.30 m, with the upper rim extending 0.03 m above the soil surface. The soil used was black soil, collected from the plow layer of a maize field in Northeast China in 2015. The baseline physicochemical properties were: organic carbon (SOC) 21.53 g kg^−1^, total nitrogen (TN) 1.46 g kg^−1^, total phosphorus (TP) 0.65 g kg^−1^, total potassium (TK) 15.80 g kg^−1^, nitrate nitrogen (NO_3_^−^-N) 48.20 mg kg^−1^, ammonium nitrogen (NH_4_^+^-N) 16.90 mg kg^−1^, available phosphorus (AP) 50.44 mg kg^−1^, and available potassium (AK) 170.00 mg kg^−1^.

### 4.2. Experimental Treatments

**Figure 8:**
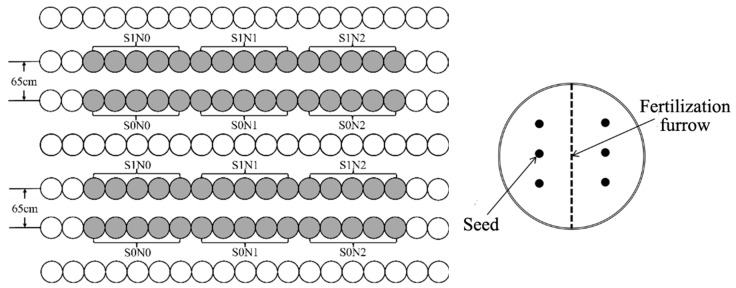
Field layout and planting pattern. Note: Open circles represent guard rows; solid circles represent treatment rows.

The experiment utilized a factorial design with two straw application levels and three nitrogen (N) fertilization rates. Straw treatments included no straw return (S0, 0 g frame^−1^ and straw return (S1, 84 g frame^−1^, equivalent to 12.0 t ha^−1^ full field return). Nitrogen fertilizer was applied as a single basal application at three levels: no N (N0), medium N (N1), and high N (N2). The experiment consisted of 10 replicates per treatment, totaling 60 micro-plots. For the N1 treatment, 1.24 g of urea was applied per frame (equivalent to 175 kg ha^−1^), while the N2 treatment received 2.47 g of urea per frame (equivalent to 350 kg ha^−1^). In both N1 and N2 treatments, one-third of the annual urea input was replaced with ^15^N-labeled urea (10.08% abundance), resulting in a final mixture abundance of 3.603%. Additionally, all treatments received calcium superphosphate (1.06 g frame^−1^, 44% P_2_O_5_, equivalent to 150 kg ha^−1^) and potassium sulfate (1.06 g frame^−1^, 50% K_2_O, equivalent to 150 kg ha^−1^) as basal fertilizers. The layout of the fields and planting patterns are shown in Figure 8. Maize seeds were sown in shallow holes (6 seeds per frame) and covered with 3–4 cm of soil. Following seedling emergence, plants were thinned to four per frame. Destructive sampling was subsequently conducted as follows: two plants at the seedling stage, one at the jointing stage, and one at the maturity stage.

### 4.3. Sampling Methods

- Plant Sampling:

Plant samples were collected on sunny mornings at the three-leaf (V3), jointing (V6), and physiological maturity (R6) stages. The aboveground biomass was harvested by cutting at the soil surface. Samples were heat-treated at 105 °C for 30 min to plant fixation, followed by oven-drying at 85 °C to a constant weight. The dried samples were then weighed, ground to a fine powder, and stored for subsequent analysis.

- Soil Sampling:

Soil samples were collected after the autumn harvest using a 3.0 cm diameter soil drill from two depths: 0–15 cm (topsoil) and 15–30 cm (subsoil). Approximately 300 g of fresh soil was composited from five randomly selected micro-plots per treatment. Visible plant roots and organic debris were manually removed. The samples were then divided: 100 g was stored at −20 °C for fresh soil analysis, and the remaining portion (approx. 100 g) was air-dried, ground, and sieved for analysis.

### 4.4. Measurement Indices and Methods

- Dry weight of plant parts (aboveground biomass, cob, and grain):

Determination of plant nitrogen content and ^15^N abundance: Plant nitrogen content was first determined using the Kjeldahl method. The titrated solution was then concentrated and reacted with lithium hypobromite (LiOBr) under cryogenic vacuum conditions to generate N_2_ gas. The ^15^N abundance was measured using a Delta V Advantage Isotope Ratio Mass Spectrometer (Thermo Fisher Scientific, USA) in dual-inlet (DI) mode [37].

- Soil N-transforming enzyme activities:

Determination of soil nitrate reductase (S-NR) and nitrite reductase (S-NiR) activities: Sulfanilic acid-α-naphthylamine colorimetric method.

Determination of soil urease (S-UE) activity: Sodium phenate-sodium hypochlorite colorimetric method.

- Determination of physiological and biochemical indicators:

Plant total nitrogen determination: H_2_SO_4_-H_2_O_2_ digestion followed by the Kjeldahl method [38].

Plant total phosphorus determination: Concentrated H_2_SO_4_ digestion followed by molybdenum-antimony anti-spectrophotometry [38].

Plant total potassium determination: H_2_SO_4_-H_2_O_2_ digestion followed by flame photometry [38].

Soil total nitrogen determination: H_2_SO_4_ digestion followed by the Kjeldahl method (Buchi K350, Switzerland) [37].

Yield components: Cob length, cob diameter, cob weight, and grain number per cob were measured. The 100-grain weight was determined by weighing 100 randomly selected uniform kernels per ear.

Maize yield determination: Yield was calculated by weighing the kernels after threshing.

### 4.5. Data Analysis

Data processing and statistical analyses were performed using Microsoft Excel 2016 and SPSS 21.0 (SPSS Inc., Chicago, IL, USA). Prior to statistical testing, all data were checked for normality. Treatment means were compared using one-way analysis of variance (ANOVA) followed by Duncan’s multiple range test at the *p* < 0.05 significance level.

### 4.6. Calculations

Grain number per cob = Kernel rows per ear × Kernels per row.

Plant N accumulation (mg/plant) = Plant N concentration (g/kg) × Plant dry matter weight (g).

Plant P accumulation (mg/plant) = Plant P concentration (g/kg) × Plant dry matter weight (g).

Plant K accumulation (mg/plant) = Plant K concentration (g/kg) × Plant dry matter weight (g).

Percentage of nitrogen derived from fertilizer (Ndff, %):

$$\mathrm{Ndff}(\%)\;=\;\frac{\left(f_{sample}-f_{natural}\right)}{\left(f_{fertilizer}-f_{natural}\right)}\times 100$$

where $f_{sample}$ is the ^15^N abundance of the plant sample, $f_{natural}$ is the natural ^15^N abundance (0.3663%), and $f_{fertilizer}$ is the ^15^N abundance of the labeled fertilizer.

Nitrogen use efficiency (NUE, %):

$$\mathrm{NUE}(\%)\;=\;\frac{Plant\;N\;accumulation\times Ndff(\%)}{N\;aoolication\;amount(g)}$$

## 5. Conclusions

The integration of straw return with appropriate nitrogen (N) supplementation bolsters maize yield by enhancing crop nutrient uptake capacity and optimizing soil N cycling functions; applying optimal N fertilizer subsequently stimulates the release of indigenous soil nitrogen, facilitating the continuous uptake of nutrients from the soil by the maize plants. Under N-deficient conditions, straw return acts primarily as a restrictive factor. Conversely, when coupled with rational N application, straw return facilitates plant nutrient absorption and soil N transformation capacity, ultimately achieving a concurrent elevation in nutrient accumulation, fertilizer use efficiency, and grain yield.

## Figures and Tables

**Table 1:** Content of soil total nitrogen in corn-cultivated soil (g/kg).

Year	Treatment		Deep (0–15 cm)	Deep (15–30 cm)
2023	S0	N0	1.11 ± 0.02 d	1.14 ± 0.05 c
		N1	1.2 ± 0.03 c	1.24 ± 0.05 ab
		N2	1.22 ± 0.03 c	1.29 ± 0.06 ab
	S1	N0	1.37 ± 0.03 b	1.15 ± 0.07 c
		N1	1.45 ± 0.02 a	1.37 ± 0.06 a
		N2	1.48 ± 0.02 a	1.42 ± 0.06 a
2024	S0	N0	1.24 ± 0.04 c	1.17 ± 0.03 c
		N1	1.23 ± 0.03 c	1.21 ± 0.02 c
		N2	1.22 ± 0.03 c	1.23 ± 0.09 bc
	S1	N0	1.53 ± 0.05 b	1.23 ± 0.04 bc
		N1	1.61 ± 0.02 ab	1.36 ± 0.05 ab
		N2	1.63 ± 0.02 a	1.42 ± 0.02 a

Note: Data are presented as means ± standard error (*n* = 4). Different lowercase letters within the same column indicate significant differences between treatments at *p* < 0.05.

**Table 2:** Nitrogen-transforming enzyme activity in corn-cultivated soil in 2023.

Deep (cm)	Treatment		Soil Nitrate Reductase Activity (μmol/d/g)	Soil Nitrite Reductase Activity (μmol/d/g)	Soil Urease Activity (μg/d/g)
0–15	S0	N0	0.37 ± 0.01 d	6.07 ± 0.08 e	460.91 ± 6.98 d
		N1	0.57 ± 0.00 c	7.03 ± 0.31 d	482.82 ± 37.39 cd
		N2	0.79 ± 0.06 b	8.62 ± 0.07 bc	707.09 ± 8.33 b
	S1	N0	0.97 ± 0.07 a	8.09 ± 0.17 c	662.92 ± 40.99 b
		N1	0.99 ± 0.06 a	9.21 ± 0.34 ab	631.01 ± 115.45 bc
		N2	1.05 ± 0.03 a	9.78 ± 0.24 a	952.06 ± 3.69 a
15–30	S0	N0	0.24 ± 0.05 d	5 ± 0.15 c	455.37 ± 6.79 d
		N1	0.52 ± 0.04 cd	6.13 ± 0.35 bc	481.76 ± 94.81 cd
		N2	0.62 ± 0.06 bc	7.3 ± 0.29 b	647.87 ± 10.48 b
	S1	N0	0.65 ± 0.23 bc	6.75 ± 0.44 b	510.78 ± 14.12 bcd
		N1	0.87 ± 0.04 ab	9.16 ± 0.12 a	614.4 ± 45.81 bc
		N2	0.99 ± 0.02 a	8.98 ± 0.64 a	807.45 ± 39.7 a

Note: Data are presented as means ± standard error (*n* = 4). Different lowercase letters within the same column indicate significant differences between treatments at *p* < 0.05.

**Table 3:** Nitrogen-transforming enzyme activity in corn-cultivated soil in 2024.

Deep (cm)	Treatment		Soil Nitrate Reductase Activity (μmol/d/g)	Soil Nitrite Reductase Activity (μmol/d/g)	Soil Urease Activity (μg/d/g)
0–15	S0	N0	0.43 ± 0.04 b	4.51 ± 0.3 d	529.77 ± 28.35 d
		N1	0.53 ± 0.03 b	6.19 ± 0.28 cd	606.3 ± 44.96 cd
		N2	0.91 ± 0.15 a	7.48 ± 0.42 abc	715.67 ± 59.04 abc
	S1	N0	0.84 ± 0.08 a	6.61 ± 0.33 bcd	650.93 ± 38.7 bcd
		N1	1.14 ± 0.02 a	8.29 ± 0.25 ab	767.33 ± 72.71 ab
		N2	1.01 ± 0.08 a	8.45 ± 0.19 a	843.84 ± 11.58 a
15–30	S0	N0	0.19 ± 0.03 c	4.24 ± 0.16 c	215.72 ± 14.02 c
		N1	0.51 ± 0.04 bc	5.8 ± 0.49 b	431.86 ± 77.19 b
		N2	0.54 ± 0.13 b	6.19 ± 0.65 b	499.95 ± 69.75 b
	S1	N0	0.55 ± 0.19 b	5.39 ± 0.49 ab	634.19 ± 61.33 ab
		N1	0.85 ± 0.12 ab	7.54 ± 0.66 a	726.05 ± 88.77 a
		N2	1.23 ± 0.05 a	8 ± 0.46 a	827.02 ± 33.12 a

Note: Data are presented as means ± standard error (*n* = 4). Different lowercase letters within the same column indicate significant differences between treatments at *p* < 0.05.

**Table 4:** Yield components of maize at harvest stage in 2023 and 2024.

Year	Treatment		Cob Length (cm)	Cob Diameter (mm)	Grain Number Per Spike (pcs)	Hundred Kernel Weight (g)
2023	S0	N0	16.8 ± 0.89 b	4.51 ± 0.03 b	538.67 ± 35.6 b	32.51 ± 1.64 a
		N1	17.4 ± 1.01 ab	4.8 ± 0.09 a	596.00 ± 6.11 b	33.12 ± 0.54 a
		N2	19.83 ± 0.58 a	4.92 ± 0.11 a	745.33 ± 42.35 a	31.23 ± 1.45 a
	S1	N0	12.9 ± 0.60 b	4.74 ± 0.07 a	380.00 ± 18.04 c	37.22 ± 1.29 a
		N1	17.3 ± 1.60 a	4.72 ± 0.06 a	601.33 ± 11.39 b	33.20 ± 0.62 b
		N2	18.63 ± 0.79 a	4.68 ± 0.07 a	677.33 ± 5.33 a	32.41 ± 0.22 b
2024	S0	N0	16.77 ± 0.28 a	4.5 ± 0.05 a	546.00 ± 21.39 b	27.55 ± 0.95 c
		N1	16.2 ± 0.5 a	4.46 ± 0.16 a	434.67 ± 14.67 a	35.20 ± 0.79 b
		N2	17.37 ± 0.52 a	4.5 ± 0.05 a	447.33 ± 8.67 a	39.00 ± 0.16 a
	S1	N0	17.57 ± 0.24 a	4.29 ± 0.1 a	480.67 ± 18.99 a	30.38 ± 0.8 c
		N1	17.93 ± 0.35 a	4.6 ± 0.09 a	508.67 ± 9.33 a	36.19 ± 0.74 b
		N2	17.57 ± 0.46 a	4.47 ± 0.05 a	488.67 ± 4.67 a	39.26 ± 0.91 a

Note: Data are presented as means ± standard error (*n* = 4). Different lowercase letters within the same column indicate significant differences between treatments at *p* < 0.05.

## Data Availability

All data are included in the main text.

## Abbreviations

The following abbreviations are used in this manuscript:

NUE	Nitrogen fertilizer use efficiency
S-NR	Soil Nitrate Reductase
S-NiR	Soil nitrite reductase
S-UE	Soil urease
Ndff	Nitrogen derived from fertilizer
